# Microbial SynComs as biocontrol tools: modulating plant immunity and future perspectives

**DOI:** 10.3389/fmicb.2026.1768717

**Published:** 2026-04-10

**Authors:** Vishwas Gaur, Mandavi Gosain, Madhu Kamle, Karuna Shrivastava, Ajey Singh, Pradeep Kumar

**Affiliations:** 1 Applied Microbiology Laboratory, Department of Botany, University of Lucknow, Lucknow, India; 2 Department of Biochemistry, University of Lucknow, Lucknow, India; 3 Department of Forestry, North Eastern Regional Institute of Science and Technology, Nirjuli, Arunachal Pradesh, India; 4 Department of Food Biosciences and Technology, Korea University, Seoul, Republic of Korea

**Keywords:** biocontrol, microbial consortia, phytopathogen, soil health, sustainable agricultural

## Introduction

The evolutionarily conserved interaction between plants and their microbiota shows a dynamic coevolved system that supports plant health, production, and ecological adaptation within the holobiont (Dolatabadian, 2020; Laishram et al., 2025). Beneficial microbes serve a wide range of important functions in plant systems, including atmospheric nitrogen fixation, organic residue breakdown, pesticide detoxification, disease suppression, and nutrient cycle enhancement (Wang et al., 2022). They produce bioactive chemicals such as vitamins, hormones, and enzymes, which promote plant growth and development. Furthermore, plant growth-promoting rhizobacteria (PGPR) and fungi (PGPF) reduce abiotic stress by promoting phytohormone and siderophore production, phosphate solubilization, ethylene reduction, and upregulation of dehydration-responsive and antioxidant genes, thereby increasing plant resilience and productivity (Koza et al., 2022). Despite these remarkable capabilities, consistently harnessing microbial functions in field conditions remains difficult, emphasizing the critical need for biocontrol agents (BCAs) that are stable, predictable, and effective over a wide range of situations.

BCAs are essential for organic and sustainable agriculture; however, the traditional use of single-strain inoculants, such as *Pseudomonas, Bacillus*, and *Trichoderma*, has repeatedly shown poor field performance (Tyagi et al., 2024). This constraint reflects a broader biological reality: plant immunity and disease suppression arise from complex microbial networks rather than from isolated species. As a result, there is an increasing trend toward community-based solutions. Microbiome transfer methods, such as soil transfer and rhizosphere microbiome transplants, have shown that disease suppression can be replicated by relocating complete microbial populations. However, these approaches are still hampered by soil heterogeneity and limited control over microbial composition (Jiang et al., 2022).

To address these issues, Synthetic Microbial Communities (SynComs) have evolved as a more controlled and environmentally conscious approach. Typically consisting of 2–5 well-selected taxa, such as *Bacillus, Pseudomonas, Enterobacter, Streptomyces*, or *Pantoea*. SynComs are intended to emulate the functional variety found in natural microbiomes. Importantly, their selection now prioritizes features that promote microbial fitness and plant health, such as exometabolite synthesis, VOC emission, strong biofilm formation, and the ability to control host immunity. Evidence supports this shift: Kampa et al. (2025) demonstrated that compatible *Trichoderma*-*Bacillus* consortia provide more stable and broader disease suppression than single isolates, whereas Ma et al. (2021) demonstrated that specific root commensals within SynComs can suppress MAMP-induced growth inhibition and stabilize plant immunity by fine-tuning defense gene expression. These findings reinforce that effective and predictable biocontrol will rely on well-designed microbial consortia capable of colonizing, interacting synergistically, and maintaining immune homeostasis across a wide range of environmental conditions.

## Microbial consortia and plant immunity

Plant immunity, which has evolved over millions of years, functions as a highly coordinated two-branched defensive mechanism in which surface-localized Pattern Recognition Receptors (PRRs) identify microbial patterns and trigger the first layer of innate immune protection (Zhang et al., 2023). Plant immunity is based on two major layers of defense: PTI (Pattern Triggered Immunity), which is activated when PRRs detect MAMPs, PAMPs (Pathogen-Associated Molecular Patterns), HAMPs (Herbivore-Associated Molecular Patterns), or DAMPs (Damage-Associated Molecular Patterns), and ETI (Effector-Triggered Immunity), which is triggered by intracellular NLRs (NOD-Like Receptors) that recognize pathogen effectors. PTI and ETI together activate systemic acquired resistance (SAR). Traditional biological control agents aid these defenses through direct antagonism, antibiosis, parasitism, lytic enzymes, and indirect mechanisms like competition and induced resistance (Yu et al., 2022). However, their impact is restricted since individual strains cannot fully modify the plant immune network.

SynComs improve plant immunity through coordinated, multi-tiered mechanisms that outperform single-strain biocontrol agents. SynComs function as integrated immune modulators, activating Induced Systemic Resistance (ISR), priming salicylic acid (SA), jasmonic acid (JA), and ethylene (ET) signaling pathways while fine-tuning pattern-recognition-based immunological perception (Jung et al., 2024). This comprehensive involvement puts SynComs at the forefront of sustainable plant protection techniques. Recent evidence supports this viewpoint. Bashizi et al. (2025) revealed the immune-modulating ability of microbial consortia in pepper plants infected with *Phytophthora capsici*. A five-strain SynCom derived from various rhizobacterial genera not only reduced disease severity but also increased plant growth via phosphate solubilization, siderophore synthesis, and high antipathogen activity. Importantly, the SynCom treatment changed the rhizosphere microbiome, increasing beneficial taxa like *Bacillus* and *Trichoderma* while also promoting functions related to nutrient cycling and disease control.

**Figure 1:**
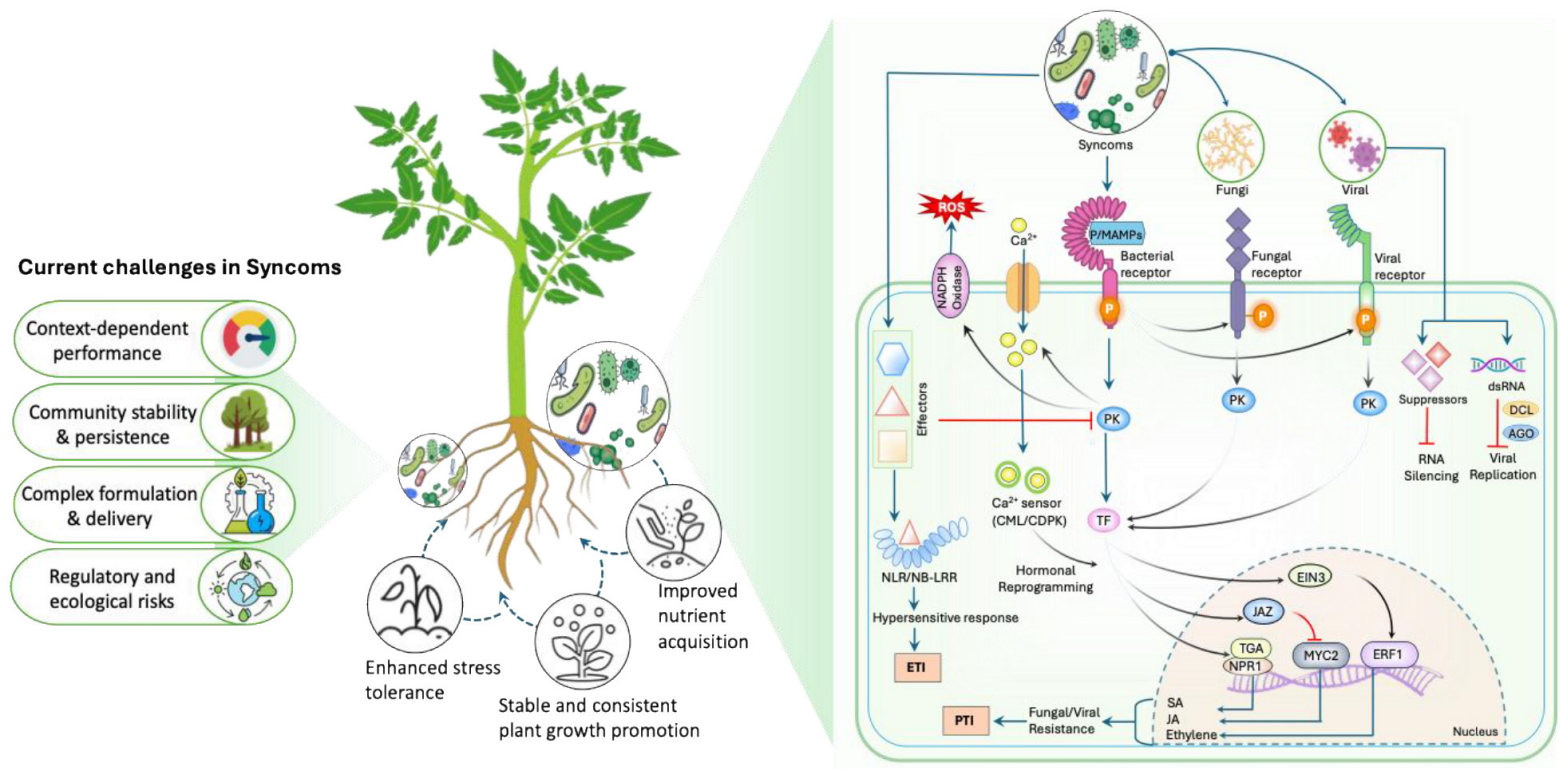
SynCom-mediated regulation of plant immunity showing modulation of PTI and ETI through pathogen recognition receptors, activation of phosphorylation-driven kinase cascades, and induction of SA-, JA-, and ET-responsive genes, collectively promoting plant growth and development; key challenges of current SynCom applications are illustrated on the right.

Beneficial bacteria can stimulate plant immunity by triggering ISR and priming, allowing plants to respond more quickly and efficiently to pathogens. SynComs improve this impact by mixing various synergistic microorganisms that promote long-distance signaling and overall immunological preparedness (Yu et al., 2022). SynComs also affect local host immunity by inhibiting flg22-triggered MTI (MAMP-Triggered Immunity) and reversing root development inhibition. Beneficial bacteria accomplish this through a variety of ways, including reducing rhizosphere pH through gluconic acid synthesis, decomposing auxin to regulate root hormone levels, and directly degrading the flg22 peptide (Martins et al., 2023; Teixeira et al., 2021). Aside from lowering local defense costs, SynComs also stimulate critical hormonal pathways such as SA, JA, and ET in ways that solo inoculants rarely do (Figure 1). For example, Gupta et al. (2022) found that a SynCom enriched with cytokinin-responsive *Bacillus* strains (*B. megaterium* and *B. subtilis*) induced substantial ISR in tomato via activating SA, JA, and ET signaling, although having little direct antibacterial action. In contrast, Gram-negative isolates elicited significantly lesser responses. These findings demonstrate the ability of rationally constructed, synergy-driven SynComs to reprogram plant immune networks more stably and durably than traditional biocontrol techniques. However, because immune activation is often linked to growth penalties, SynCom-based strategies should explicitly address immune–growth trade-offs, favoring immune priming over constitutive defense activation to achieve durable protection without compromising plant growth and yield.

## Discussion

The potential of SynComs stems from their ability to mimic ecologically relevant microbial functions that single-strain inoculants cannot; yet, this promise is coupled with significant complexity. High-diversity SynComs may offer functional redundancy, increased robustness, microbial antagonist suppression, and the development of favorable microenvironments (Cambon et al., 2025). However, these benefits are not without drawbacks: as species diversity grows, so does the number of biotic interactions, making it increasingly difficult to anticipate SynCom behavior, stability, and compatibility with the host plant. This ecological complexity underlines a key difficulty for the field: how to design SynComs that maintain robustness while minimizing unwanted interactions that could reduce their effectiveness or affect plant responses in unexpected ways (Dubey et al., 2025).

Given these constraints, it is crucial in SynCom research to critically connect the community design with the biological questions being answered, while also explicitly addressing the limitations inherent in any model system. Rigorous validation of SynCom performance, whether through assessments of community composition, development, interactions, or host and ecological responses, is critical for generating meaningful insights. Researchers can now evaluate both structural and functional attributes of SynComs simultaneously thanks to strain barcoding and multi-omics technologies; however, depending on whether the study focuses on ecological dynamics or functional outputs, comprehensive dual validation is not always required (Mehlferber et al., 2024; Ordon et al., 2024).

The next generation of SynComs should be considered as dynamic immunological partners that interact with and strengthen plant defense networks, rather than as basic biological inputs. This vision necessitates an integrated approach that combines plant immunity, microbial ecology, systems biology, and predictive modeling to create communities with functional resilience, ecological stability, and tailored immune-modulating capability. Moving forward, an immunity-centric and ecology-informed paradigm is required, one that recognizes the complexities of plant-microbiome interactions and uses rigorous validation to ensure reliability. As SynComs transition from lab systems to real-world agricultural applications, ethical problems become critical. Ethical SynCom development entails guaranteeing environmental safety, minimizing harm to native microbiomes, and preventing unintentional invasions. High-density applications necessitate thorough risk assessment, adherence to key ethical standards, and enhanced biocontainment to guarantee responsible and sustainable usage in agricultural settings (Mehlferber et al., 2024). Although SynComs are frequently promoted as alternatives to genetically modified organisms (GMOs), rationally designed or genetically optimized consortia can pose similar concerns related to environmental release, horizontal gene transfer, ecological persistence, and unintended impacts on native microbiomes. Therefore, clear regulatory frameworks, standardized risk-assessment protocols, and transparency in strain selection and functional trait design are essential to ensure public trust and the responsible, sustainable adoption of SynCom technologies in agriculture.

We propose that future SynCom research should shift from static, taxonomically defined consortia toward function-driven, context-responsive assemblies that dynamically adapt to host genotype, soil type, and environmental stress. We further hypothesize that inter-microbial interactions and emergent metabolic complementarity, rather than individual strain performance, represent the primary determinants of SynCom stability and field efficacy. Framing these ideas as clear hypotheses will not only distinguish the authors' conceptual contribution but also provide a focused roadmap for experimental validation and translational application.
